# Direct Ophthalmic Artery Puncture for Coil Embolization of a Traumatic Internal Carotid Artery Pseudoaneurysm With a Concomitant Carotid-Cavernous Fistula Following a Transorbital Penetrating Injury

**DOI:** 10.7759/cureus.103497

**Published:** 2026-02-12

**Authors:** Riku Ohwada, Yasuhiro Takahashi, Sangnyon Kim, Takeshi Mikami, Nobuhiro Mikuni

**Affiliations:** 1 Department of Neurosurgery, Sapporo Medical University, Sapporo, JPN; 2 Department of Neurosurgery, Sapporo City General Hospital, Sapporo, JPN

**Keywords:** carotid–cavernous fistula (ccf), ophthalmic artery direct puncture, pseudoaneurysm, transorbital penetrating injury, traumatic internal carotid artery pseudoaneurysm (tica)

## Abstract

The coexistence of a traumatic internal carotid artery (ICA) pseudoaneurysm and a carotid-cavernous fistula (CCF) after a transorbital penetrating injury is rare and presents major therapeutic challenges. We report a case of a Denver Grade III traumatic ICA pseudoaneurysm (TICA) with longitudinal arterial dissection, for which reconstructive endovascular treatment was unsuitable; therefore, bypass-assisted ICA trapping was selected as the initial management. Despite parent artery occlusion, postoperative angiography revealed persistent pseudoaneurysmal filling via retrograde collateral inflow from the external carotid system through the ophthalmic artery. The traumatic CCF became angiographically evident after ICA trapping, indicating altered hemodynamics. Both transvenous embolization via the inferior petrosal sinus and retrograde transarterial access through the facial and angular arteries were attempted but failed because of anatomical constraints. As standard strategies could not achieve complete exclusion, controlled surgical access to the ophthalmic artery was selected as the salvage approach. Using the outer cannula of an 18-gauge intravenous catheter as a sheath substitute, a microcatheter was advanced into the pseudoaneurysm, and coil embolization was performed. Complete obliteration of both the pseudoaneurysm and the CCF was achieved without additional neurological deficits. This case demonstrates that direct ophthalmic artery access may serve as a highly selective salvage option in exceptional circumstances when TICA and CCF coexist and conventional approaches are not feasible.

## Introduction

Transorbital penetrating injuries can damage the intraorbital structures, skull base, and parasellar region, and may result in traumatic cerebrovascular lesions with no standardized management. Traumatic internal carotid artery pseudoaneurysms (TICAs) are rare (<1% of intracranial aneurysms) but carry a high risk of rupture, particularly when classified as Denver Grade III, requiring prompt intervention [1-4]. These lesions are often accompanied by longitudinal internal carotid artery (ICA) dissection, which may limit the feasibility of reconstructive endovascular treatment and necessitate parent artery occlusion or trapping.

Traumatic carotid-cavernous fistulas (CCFs) are high-flow shunts that can develop after head trauma and markedly alter cavernous sinus hemodynamics [5,6]. When a TICA coexists with a traumatic CCF, the pseudoaneurysm may function as part of the shunt pathway, requiring careful control of inflow and outflow and diminishing the effectiveness of standard embolization or trapping strategies [7].

In the present case, despite bypass-assisted ICA trapping for a Denver Grade III traumatic pseudoaneurysm, residual collateral inflow via the ophthalmic artery and emergence of a cavernous sinus CCF rendered management highly challenging. Multiple conventional endovascular approaches were unsuccessful, and repeat craniotomy was considered high-risk. Ultimately, direct puncture of the ophthalmic artery enabled definitive coil embolization with complete obliteration of both the pseudoaneurysm and the fistula. Herein, we describe the clinical course and discuss the relevance of this highly selective salvage approach.

## Case presentation

Initial assessment

**Figure 1:**
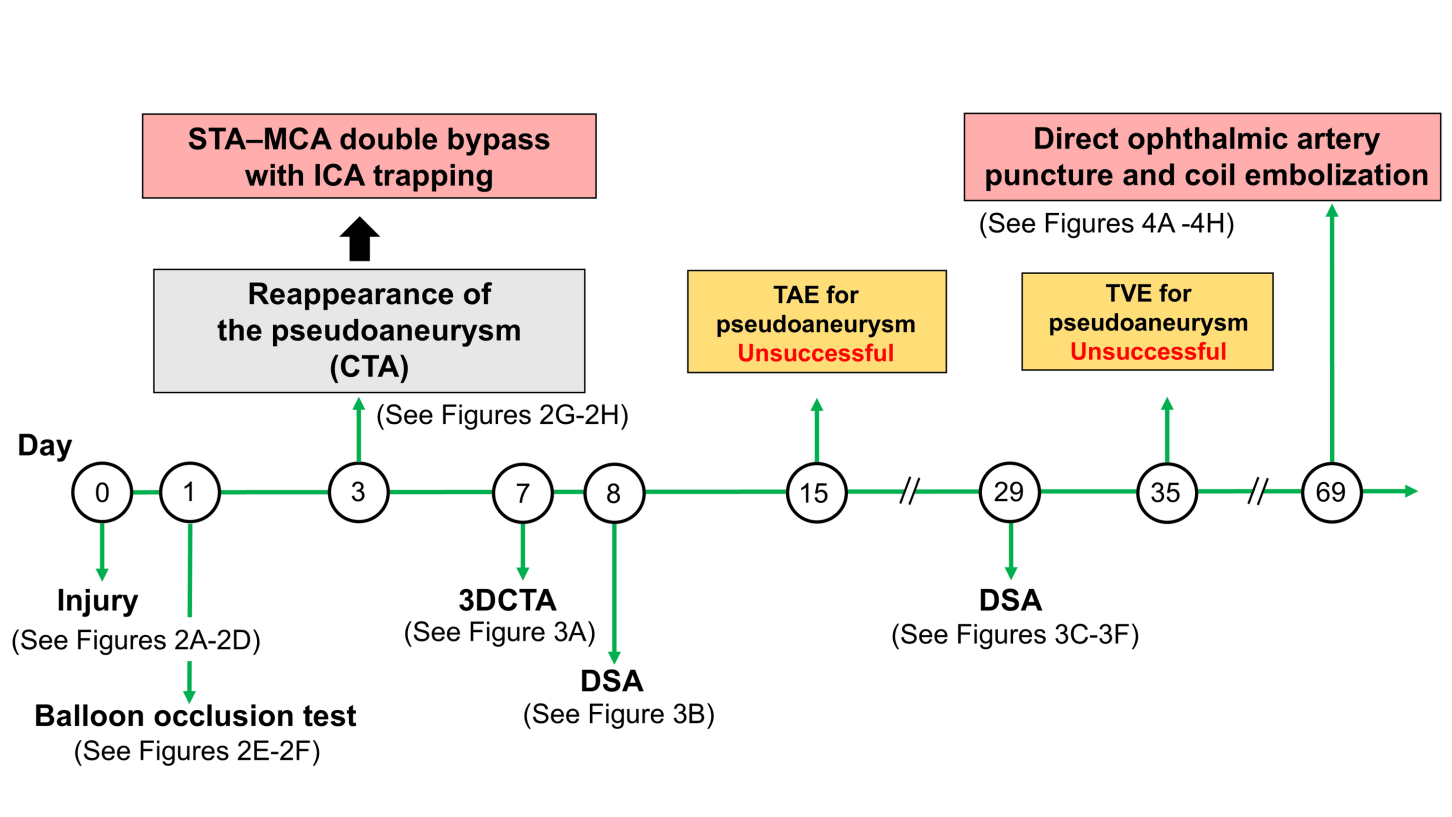
Timeline of Treatment After Injury At the time of injury, a pseudoaneurysm of the right internal carotid artery (ICA) was identified; however, it was not visualized on the balloon occlusion test performed the following day. On day 3 after the injury, computed tomography angiography (CTA) revealed reappearance of the pseudoaneurysm, prompting an emergent double superficial temporal artery-middle cerebral artery (STA-MCA) bypass and ICA trapping. On days 7 and 8, CTA and digital subtraction angiography (DSA) demonstrated retrograde flow into the aneurysm via the ophthalmic artery, indicating a carotid-cavernous fistula (CCF). Transarterial embolization (TAE) was initially attempted on day 15; however, access to the pseudoaneurysm could not be achieved. Follow-up DSA performed on day 29 revealed an overt shunt flow from the pseudoaneurysm to the cavernous sinus. Subsequent transvenous embolization (TVE) was attempted on day 35; however, catheterization of the pseudoaneurysm was unsuccessful. On day 69, coil embolization was performed via direct puncture of the ophthalmic artery.

**Figure 2:**
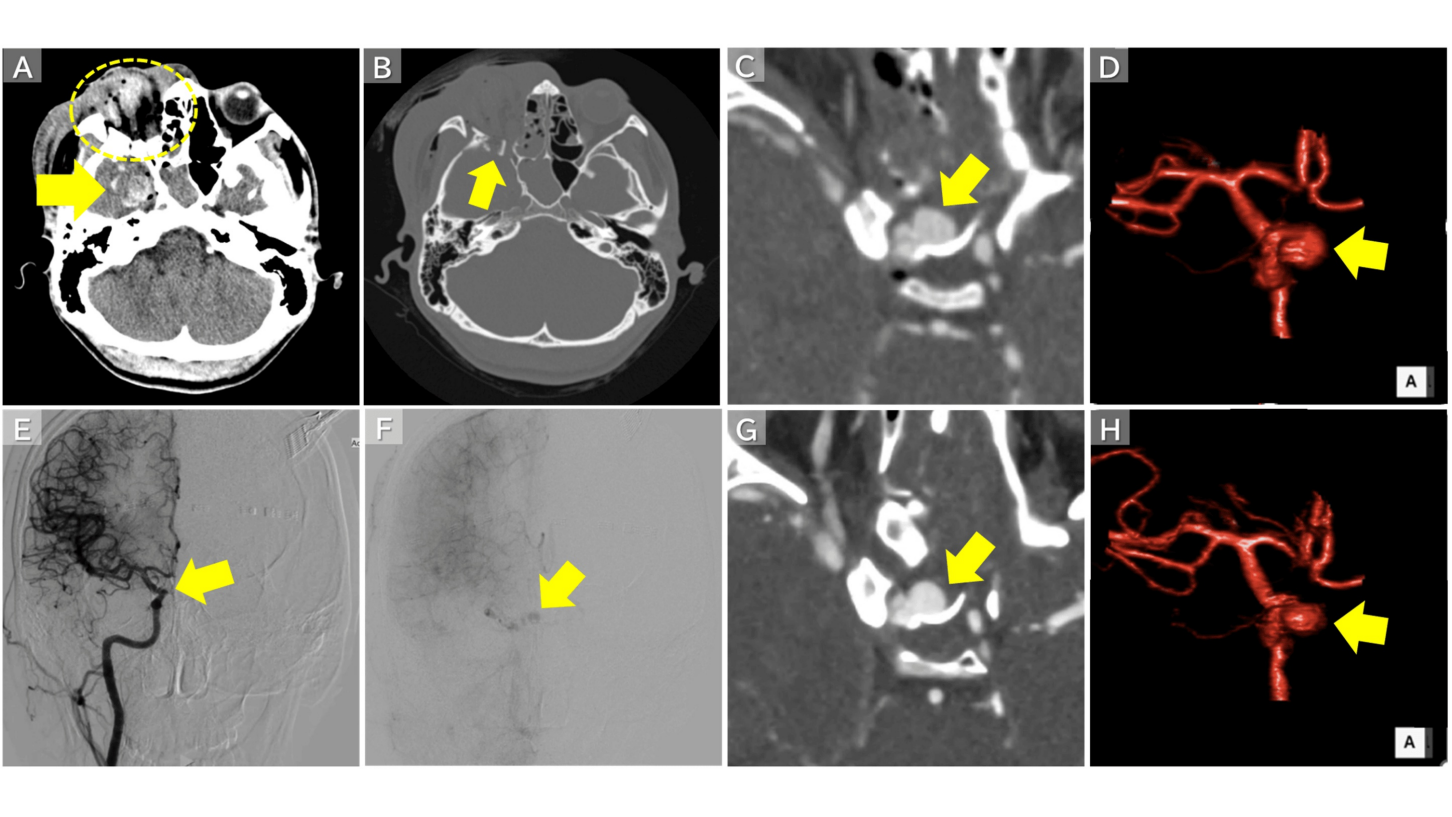
Imaging Findings Prior to Double STA-MCA Bypass and ICA Trapping (A) Right globe rupture (circle) and right temporal lobe contusion (arrow). (B) Skull base fracture (arrow). (C, D) Pseudoaneurysm of the cavernous segment of the right ICA (arrow). (E) The pseudoaneurysm was not visualized on the balloon occlusion test performed the day after injury (arrow). (F) Very faint opacification of the pseudoaneurysm was observed in the late phase. (G, H) CTA on day 3 after injury demonstrated reappearance of the pseudoaneurysm (arrow). STA-MCA: superficial temporal artery-middle cerebral artery; ICA: internal carotid artery; CTA: computed tomography angiography

The patient was a 28-year-old man who sustained a transorbital penetrating injury when a metal bar struck his right eye and penetrated intracranially (Figure 1). On arrival, his Glasgow Coma Scale score was 14 (E4V4M6), and he had a right eyelid laceration and globe rupture. Head computed tomography (CT) revealed right globe rupture, skull base fractures, and contusion of the right temporal lobe (Figures 2A-2B). CT angiography (CTA) demonstrated a pseudoaneurysm of the right cavernous ICA with extensive dissection extending from the C4 to C2 segments, consistent with Denver Grade III (Figures 2C-2D).

Balloon occlusion testing

Balloon occlusion testing was performed 1 day after injury using a 5.2-Fr Selecon MP balloon catheter (Terumo Corporation, Tokyo, Japan). Diagnostic digital subtraction angiography (DSA) before balloon inflation showed no opacification of the pseudoaneurysm, even in the late phase (Figure 2E). However, subtle late-phase opacification became apparent on DSA obtained after balloon inflation (Figure 2F), suggesting delayed filling through a dissected pseudolumen and dynamic lesion instability. Temporary observation was selected, considering the possibility of spontaneous thrombosis.

Bypass-assisted trapping

Repeat CTA on day 3 demonstrated the pseudoaneurysm (Figures 2G-2H). Because of extensive dissection with luminal narrowing (approximately 52.2% stenosis) and difficulty securing true-lumen access, reconstructive endovascular treatment was considered unsuitable. Bypass-assisted ICA trapping was therefore performed. A double superficial temporal artery-middle cerebral artery (STA-MCA) bypass was constructed, followed by ICA trapping proximal to the posterior communicating artery.

Residual lesion and failed endovascular approaches

**Figure 3:**
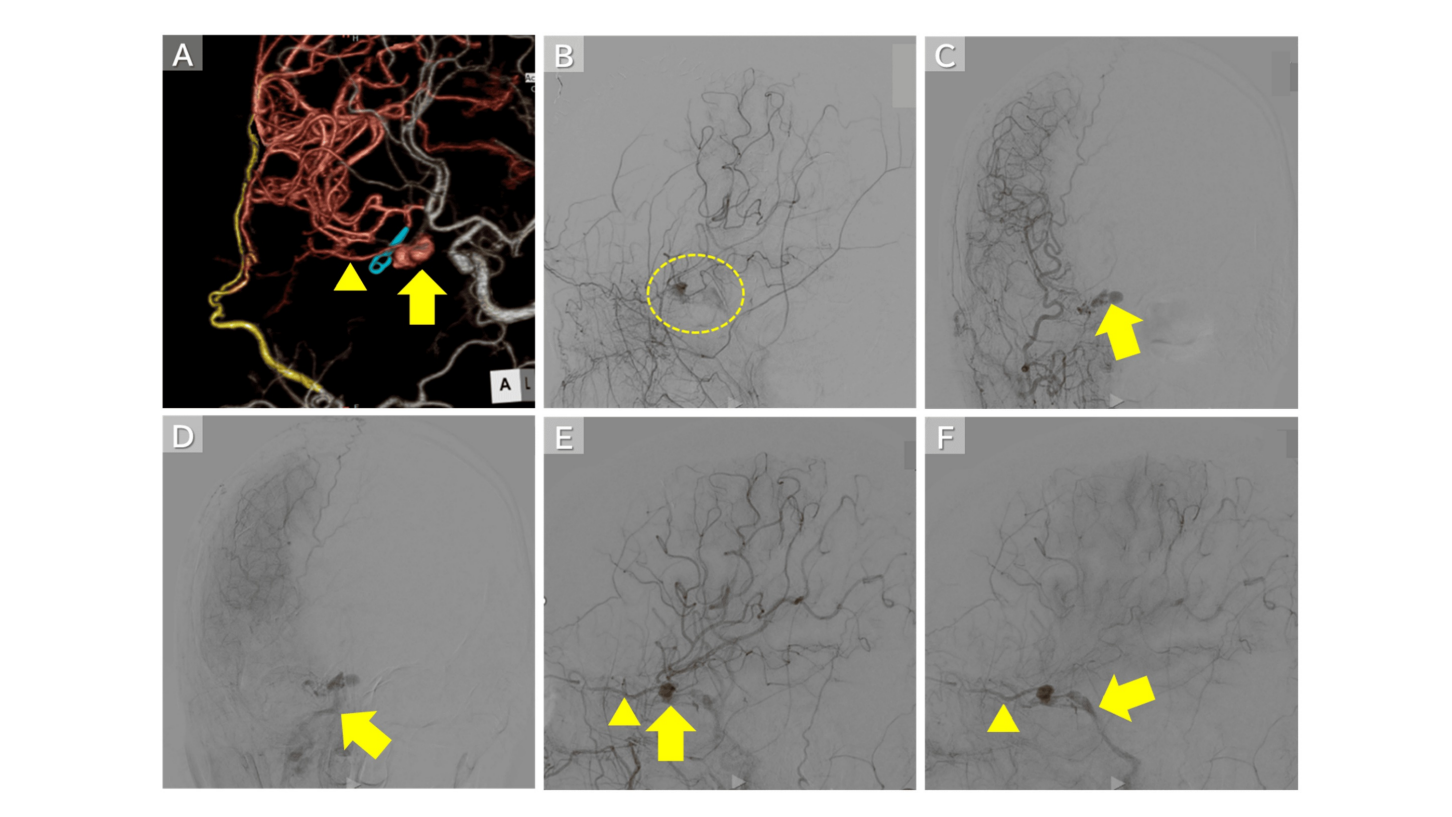
Imaging Findings After Double STA-MCA Bypass and ICA Trapping (A) CTA on postoperative day 4 showed suspected flow from the ophthalmic artery (arrowhead) into the pseudoaneurysm (arrow). (B) Cerebral angiography demonstrated retrograde filling of the pseudoaneurysm and findings suggestive of a CCF (circle). (C, E) Arterial-phase imaging revealed retrograde flow from the ophthalmic artery (arrowhead) into the pseudoaneurysm (arrow). (D, F) Venous-phase imaging showed a CCF draining into the inferior petrosal sinus (IPS) (arrow). Arrowhead indicates the ophthalmic artery. STA-MCA: superficial temporal artery-middle cerebral artery; ICA: internal carotid artery; CTA: computed tomography angiography; CCF: carotid-cavernous fistula

Despite ICA trapping, CTA on postoperative day 4 demonstrated residual opacification of the pseudoaneurysm via the ophthalmic artery (Figure 3A). Angiography confirmed residual inflow from the external carotid artery (ECA) system and revealed shunt flow into the cavernous and inferior petrosal sinuses (IPSs), consistent with a traumatic CCF (Figure 3B). On day 15, a transarterial approach was attempted by retrogradely accessing the ophthalmic artery through the ECA via the facial and angular arteries. However, the sharp bifurcation angle between the facial artery and the angular artery made catheter advancement into the aneurysm technically difficult. On day 29, follow-up cerebral angiography demonstrated further visualization of shunt flow from the pseudoaneurysm into the cavernous and IPSs (Figures 3C-3F). On day 35, a subsequent transvenous approach via the IPS was attempted; however, catheter navigation from the cavernous sinus into the aneurysm remained difficult.

Salvage procedure

**Figure 4:**
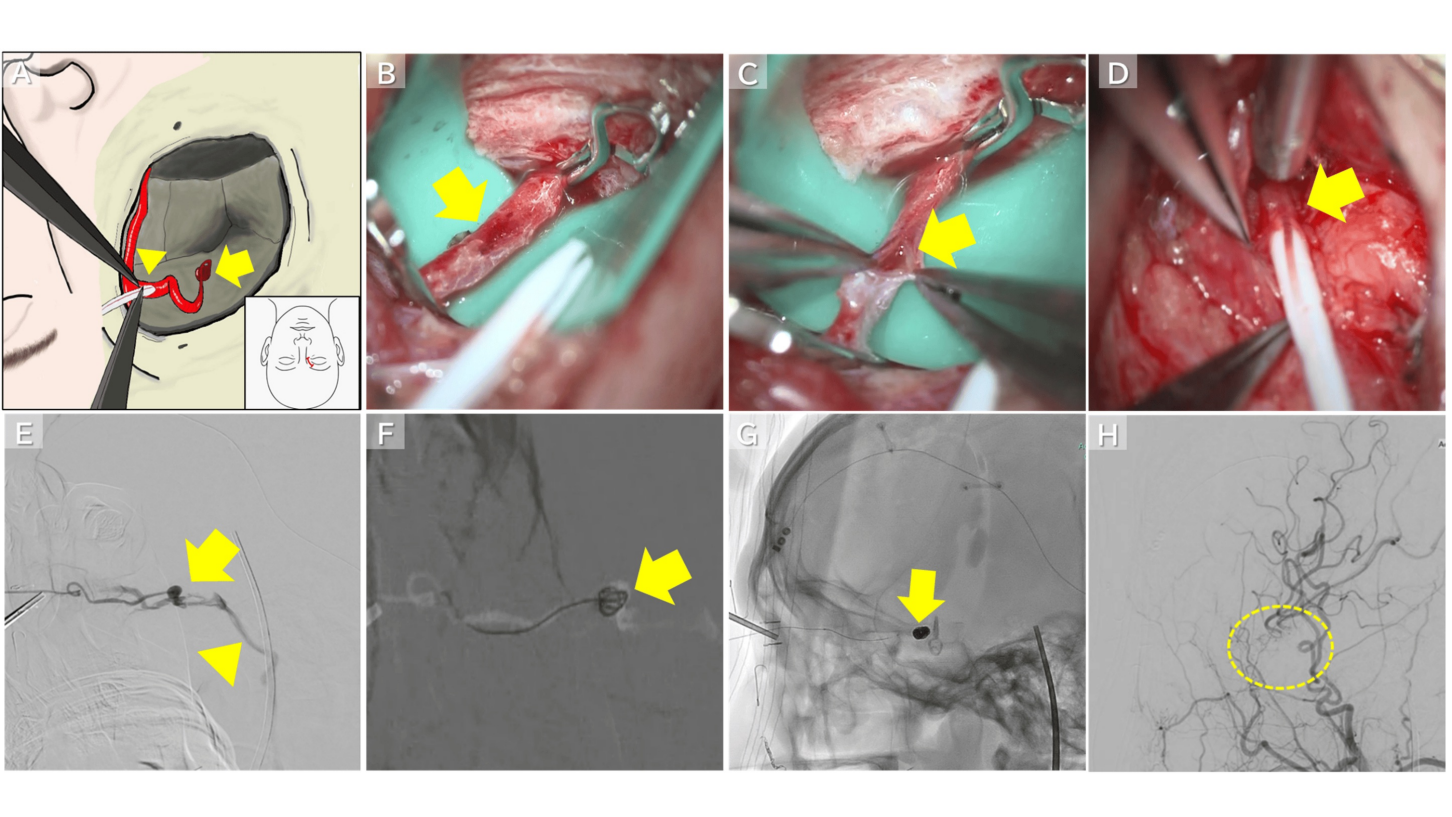
Intraoperative Findings (A) An approximately 2 cm skin incision was made in the superomedial orbit (red line). The intraorbital segment of the ophthalmic artery was incised, and a Surflo catheter was inserted (arrowhead). The arrow indicates the location of the pseudoaneurysm. Image credit: Riku Ohwada. Created using MediBang Paint. Reproduced with permission from the author. (B) The ophthalmic artery was exposed and temporarily occluded with a clip (arrow). (C) The ophthalmic artery was incised, and the arterial lumen was confirmed (arrow). (D) The ophthalmic artery was punctured, and an 18-gauge Surflo catheter outer sheath was inserted (arrow). (E) Retrograde contrast injection through the outer sheath demonstrated flow from the ophthalmic artery to the pseudoaneurysm (arrow) and into the IPS (arrowhead). (F, G) A microcatheter was inserted through the outer sheath, and coil embolization was performed (arrow). (H) Postoperative angiography showed the disappearance of the pseudoaneurysm and the CCF (circle). IPS: inferior petrosal sinus; CCF: carotid-cavernous fistula

Because the patient had completely lost visual function in the right eye due to globe rupture at the time of injury, direct arterial access via the ophthalmic artery was considered acceptable as a last-resort approach to reach the aneurysm. On day 69, the procedure was performed in a hybrid operating room equipped to allow both open microsurgical procedures and endovascular intervention. An approximately 2 cm skin incision was made in the superomedial aspect of the right orbit (Figure 4A), and the ophthalmic artery was identified and exposed under microscopic visualization (Figure 4B).

The access point was selected at the intraorbital segment (third portion) of the ophthalmic artery, immediately proximal to the origin of the dorsal nasal branch, based on arterial caliber and the feasibility of secure cannulation. After microsurgical exposure, a small arteriotomy was created under direct vision (Figure 4C), and the outer cannula of an 18-gauge Surflo intravenous catheter (Terumo Corporation) was inserted into the artery and secured as a temporary access sheath (Figure 4D).

Because the ophthalmic artery had a small caliber, placement of a conventional vascular sheath was not feasible. Therefore, the outer cannula of the 18-gauge Surflo catheter was used as the smallest practical sheath substitute that allowed safe microcatheter insertion. The inner diameter of the cannula (approximately 0.95 mm) was sufficient to accommodate a microcatheter. Although straightening of the physiological loop of the distal intraorbital segment was attempted to improve catheter alignment, limited arterial mobility prevented this maneuver, and embolization was performed with the vessel in its original configuration.

An Excelsior SL-10 microcatheter (Stryker, Kalamazoo, MI, USA) was advanced through the ophthalmic artery into the pseudoaneurysm, and coil embolization was performed (Figures 4E-4G). Final angiography confirmed complete obliteration of both the pseudoaneurysm and the CCF, with no new neurological complications (Figure 4H).

Outcome

The patient was discharged without neurological deficits other than right-eye blindness. At discharge, he was independent in activities of daily living (modified Rankin Scale score of 1). Desmopressin acetate (240 μg/day) and hydrocortisone (15 mg/day) were continued for diabetes insipidus secondary to pituitary injury, together with aspirin (100 mg/day), lacosamide (100 mg/day), and levothyroxine sodium (50 μg/day). No recurrence was observed during two years of follow-up.

## Discussion

TICAs are characterized by fragile wall structures and rarely undergo spontaneous thrombosis [1,2]. In contrast, traumatic CCFs generate high-flow arteriovenous shunts within the cavernous sinus and markedly alter intracranial venous drainage [5-7]. When these lesions coexist, the pseudoaneurysm may function not as a blind-ending sac but as part of a continuous shunt circuit with both arterial inflow and venous outflow, resulting in complex hemodynamics that complicate treatment [7-11].

The novelty of this case lies specifically in the therapeutic strategy. Direct puncture of the ophthalmic artery was considered only after bypass-assisted ICA trapping had been completed and residual pseudoaneurysmal flow persisted. Thus, this approach was not employed as a primary treatment, but strictly as a salvage option after definitive parent artery occlusion failed to achieve complete exclusion of the lesion. Notably, the CCF was not clearly evident on preoperative angiography and became apparent only after ICA trapping. This suggests that post-occlusion hemodynamic alterations may unmask a previously occult fistulous connection or augment shunt flow through a traumatic defect. Consequently, the pseudoaneurysm continued to function as part of a high-flow shunt circuit, receiving arterial inflow and providing venous outflow, which likely prevented spontaneous thrombosis. Accordingly, such potential hemodynamic alterations should be kept in mind when selecting parent artery trapping as the initial treatment strategy in similar traumatic cases.

This interpretation is consistent with prior reports indicating that TICAs associated with CCFs should be regarded as components of a single hemodynamic unit rather than independent lesions [8-11]. Duangprasert et al. reported persistent retrograde ophthalmic artery inflow after ICA trapping in a patient with a supraclinoid pseudoaneurysm and direct CCF, necessitating additional treatment [12]. Similarly, Arai et al. described aneurysmal reperfusion due to retrograde ophthalmic artery collateral flow after trapping, resulting in recurrent massive epistaxis [13].

Direct puncture of the ophthalmic artery is generally contraindicated because of the risk of catastrophic visual impairment and other serious complications, including orbital hemorrhage, arterial rupture, and thromboembolism. In this patient, however, visual function had already been irreversibly lost due to globe rupture at the time of injury. This unique clinical condition eliminated the concern for further visual deterioration and permitted acceptance of procedural risks that would otherwise be unacceptable. To our knowledge, no prior reports have described direct ophthalmic artery puncture as an arterial access route for embolization of a traumatic pseudoaneurysm or treatment of a CCF, underscoring the exceptional nature of this case.

Alternative treatment options were carefully considered. Under these complex hemodynamic conditions, standard endovascular access can be particularly challenging [7,14]. In the present case, both a transvenous approach via the IPS and a retrograde transarterial approach from the ECA were attempted; however, catheter navigation into the pseudoaneurysm proved infeasible because of severe vessel tortuosity and trauma-related vascular injury.

Repeat direct surgical treatment was also deemed high risk because adequate exposure of the ophthalmic artery was anticipated to be difficult based on the findings at the initial craniotomy, there was a substantial risk of injury to the existing STA-MCA bypass during re-exploration, and there was significant concern for uncontrolled bleeding from the cavernous sinus in the presence of an active fistula.

Endovascular alternatives were also limited. Flow diverter placement was not feasible because the ICA had already been trapped, and its acute use for traumatic pseudoaneurysms remains unsupported by sufficient evidence in Japan. A superior ophthalmic vein approach was considered [14]; however, reliable catheterization of the pseudoaneurysm and complete obliteration of the entire shunt pathway could not be ensured.

From a clinical perspective, this case demonstrates that direct puncture of the ophthalmic artery may serve as a salvage access route in extremely selected circumstances, specifically when visual function is already lost and no other surgical or endovascular strategies are feasible. The clinical value of this technique lies not in its general applicability but in its potential role as a last-resort option for otherwise untreatable lesions.

This report has important limitations. Evidence regarding the safety and efficacy of direct ophthalmic artery puncture is extremely limited, and its applicability in patients with preserved visual function remains unknown. Furthermore, the technique carries inherent risks related to arterial injury and access instability, particularly in trauma-related vessels. As no comparative studies exist, further accumulation of similar cases will be necessary to better define the indications and limitations of this highly selective salvage strategy.

## Conclusions

In this case, definitive exclusion of a TICA associated with a CCF could not be achieved using standard strategies after bypass-assisted parent artery trapping. Direct puncture of the ophthalmic artery provided secure access to the lesion and enabled successful coil embolization, resulting in complete obliteration of both the pseudoaneurysm and the fistula. Although this approach is applicable only in highly selected circumstances, this case highlights direct ophthalmic artery puncture as a novel salvage strategy that may expand therapeutic options for complex traumatic cerebrovascular lesions when conventional surgical and endovascular approaches are not feasible.

## References

[1] Zheng Y, Lu Z, Shen J, Xu F (2020). Intracranial pseudoaneurysms: evaluation and management. Front Neurol.

[2] Shi Y, Gao Y, Liu Y (2021). Treatment of traumatic intracranial pseudoaneurysms: a single-center experience. Front Neurol.

[3] Kim DY, Biffl W, Bokhari F (2020). Evaluation and management of blunt cerebrovascular injury: a practice management guideline from the Eastern Association for the Surgery of Trauma. J Trauma Acute Care Surg.

[4] Biffl WL, Moore EE, Offner PJ, Brega KE, Franciose RJ, Burch JM (1999). Blunt carotid arterial injuries: implications of a new grading scale. J Trauma.

[5] Ellis JA, Goldstein H, Connolly ES Jr, Meyers PM (2012). Carotid-cavernous fistulas. Neurosurg Focus.

[6] Henderson AD, Miller NR (2018). Carotid-cavernous fistula: current concepts in aetiology, investigation, and management. Eye (Lond).

[7] Gemmete JJ, Ansari SA, Gandhi DM (2009). Endovascular techniques for treatment of carotid-cavernous fistula. J Neuroophthalmol.

[8] Mizunari T, Murai Y, Kim K, Kobayashi S, Kamiyama H, Teramoto A (2011). Posttraumatic carotid-cavernous fistulae treated by internal carotid artery trapping and high-flow bypass using a radial artery graft--two case reports. Neurol Med Chir (Tokyo).

[9] He XH, Li WT, Peng WJ, Lu JP, Liu Q, Zhao R (2014). Endovascular treatment of posttraumatic carotid-cavernous fistulas and pseudoaneurysms with covered stents. J Neuroimaging.

[10] Karanam LS, Alurkar AB, Natarajan M, Pugazhenthi B (2014). Endovascular coil occlusion of traumatic intradural aneurysm with presentation as carotid cavernous fistula. J Clin Imaging Sci.

[11] Luo CB, Teng MM, Chang FC, Lirng JF, Chang CY (2004). Endovascular management of the traumatic cerebral aneurysms associated with traumatic carotid cavernous fistulas. AJNR Am J Neuroradiol.

[12] Duangprasert G, Sukhor S, Kaewprasert T, Tantongtip D (2023). Traumatic supraclinoid internal carotid artery pseudoaneurysm associated with carotid-cavernous fistula and contralateral anterior cerebral artery pseudoaneurysm treated by surgical trapping with high-flow bypass and A3-A3 bypass: a case report and literature review. Asian J Neurosurg.

[13] Arai N, Nakamura A, Tabuse M, Miyazaki H (2017). Late-onset massive epistaxis due to a ruptured traumatic internal carotid artery aneurysm: a case report. NMC Case Rep J.

[14] Heran MK, Volders D, Haw C, Shewchuk JR (2019). Imaging-guided superior ophthalmic vein access for embolization of dural carotid cavernous fistulas: report of 20 cases and review of the literature. AJNR Am J Neuroradiol.

